# Suppressed Recombination of Sex Chromosomes Is Not Caused by Chromosomal Reciprocal Translocation in Spiny Frog (*Quasipaa boulengeri*)

**DOI:** 10.3389/fgene.2018.00288

**Published:** 2018-08-27

**Authors:** Xiuyun Yuan, Yun Xia, Xiaomao Zeng

**Affiliations:** ^1^Chengdu Institute of Biology, Chinese Academy of Sciences, Chengdu, China; ^2^University of Chinese Academy of Sciences, Beijing, China

**Keywords:** sex determination, nascent sex chromosome, reciprocal translocation, suppressed recombination, amphibians

## Abstract

Chromosome rearrangements (CRs) are perceived to be related to sex chromosome evolution, but it is a matter of controversy whether CRs are the initial causative mechanism of suppressed recombination for sex differentiation. The early stages of sex chromosome evolution in amphibians may represent intermediate states of differentiation, and if so, they potentially shed light on the ultimate cause of suppressed recombination and the role of CRs in sex chromosome differentiation. In this paper, we showed that sex determination differs among 16 populations of spiny frog (*Quasipaa boulengeri*), in which individuals have normal and rearranged chromosomes caused by reciprocal translocation. In eastern areas, without translocation, genetic differentiation between sexes was relatively low, suggesting unrestricted recombination. In comparison, in western populations that have both normal and translocated chromosomes, a male-heterogametic system and lack of X-Y recombination were identified by male-specific alleles and heterozygote excess. However, such genetic differentiation between sexes in western populations was not directly related to karyotypes, as it was found in individuals with both normal and translocated karyotypes. In the western Sichuan Basin, male-specific and translocation-specific allelic frequency distributions suggested that recombination of sex-differentiation ceased in all populations, but recombination suppression caused by translocation did not exist in some populations. Combined with phylogenetic inference, this indicated that the establishment of sex-linkage had taken place independently of reciprocal translocation, and translocation was not the ultimate cause of sex chromosome differentiation. Furthermore, comparison of the genetic diversity of alleles on Y chromosomes, X chromosomes, and autosomes in western populations showed a reduction of effective population size on sex chromosomes, which may be caused by reciprocal translocation. It indicates that, although it is not the ultimate cause of recombination suppression, reciprocal translocation may enhance sex chromosome differentiation.

## Introduction

A commonly invoked theory explaining the origin of sex chromosomes states that the appearance of a sex-determining gene would make a pair of autosomes become proto-sex chromosomes (Charlesworth et al., 2005; Bachtrog, 2013). According to this theory, the male-beneficial but female-harmful mutations would accumulate on the proto-Y chromosome through sexually antagonistic selection close to the sex-determining region. The accumulation of sexually antagonistic genes as a selective agent promotes the evolution of reduced recombination between nascent sex chromosomes (Rice, 1987; Bachtrog, 2006). Empirical studies have highlighted that recombination suppression plays a key role in the evolution of sex chromosomes, eventually leading to the differentiation of the sex chromosomes (Wright et al., 2016).

Interestingly, another model predicts that chromosome rearrangements (CRs) are also part of crucial dynamics in sex chromosome differentiation (Ohno, 1967; Miura et al., 2012). When rearrangements occurred on sex chromosomes, recombination between the X and Y chromosomes could be suppressed, which has been supported broadly by theoretical and empirical studies (Bergero and Charlesworth, 2009; Kirkpatrick, 2010). CRs could reduce gene flow and cause recombination suppression (Rieseberg, 2001), while suppressed recombination may also affect CRs (Charlesworth et al., 2005; Wright et al., 2016). Hence, whether CRs are the initial causative mechanism of suppressed recombination for sex differentiation has been a subject of debate. Since sex chromosome evolution is often accompanied by recombination suppression and CRs, it is difficult to judge which one is the original force triggering genetic sex differentiation (Charlesworth et al., 2005; Wright et al., 2016). Traditionally, some studies proposed CRs as the causative mechanism for recombination suppression of sex chromosomes (Ohno, 1967; Miura et al., 2012). However, recent studies highlighted that CRs are derived and not the cause of sex chromosome differentiation (Natri et al., 2013; Sun et al., 2017).

To understand the roles of CRs in the early stages of sex chromosome differentiation, it is very useful to investigate a sex chromosome system accompanied by CRs. Ectothermic vertebrates differ from mammals and birds with stable sex determination and highly differentiated sex chromosomes, harboring a tremendous diversity of sex determination and sex chromosomes, both within and between species (Schmid and Steinlein, 2001; Schartl et al., 2016). Especially in amphibians, more than 96% of species investigated so far do not have morphologically distinct sex chromosomes (Eggert, 2004), indicating that frequent sex chromosome turnovers or occasional recombination can limit sex chromosome degeneration (Schartl, 2004; Stöck et al., 2011; Miura, 2017). Furthermore, numerous evolutionary transitions between different systems have been reported in many frogs, with different chromosome pairs or sex determination mechanisms in different species, even intra-species and/or within populations (Miura, 2008; Rodrigues et al., 2014, 2015; Roco et al., 2015). These patterns suggested initial sex chromosome differentiation varied within closely related species or even within the same species. Such early stages of sex chromosome differentiation systems may represent intermediate states of sex chromosome evolution, and if so, they potentially shed light on the evolutionary dynamics of the ultimate cause of suppressed recombination.

**FIGURE 1:**
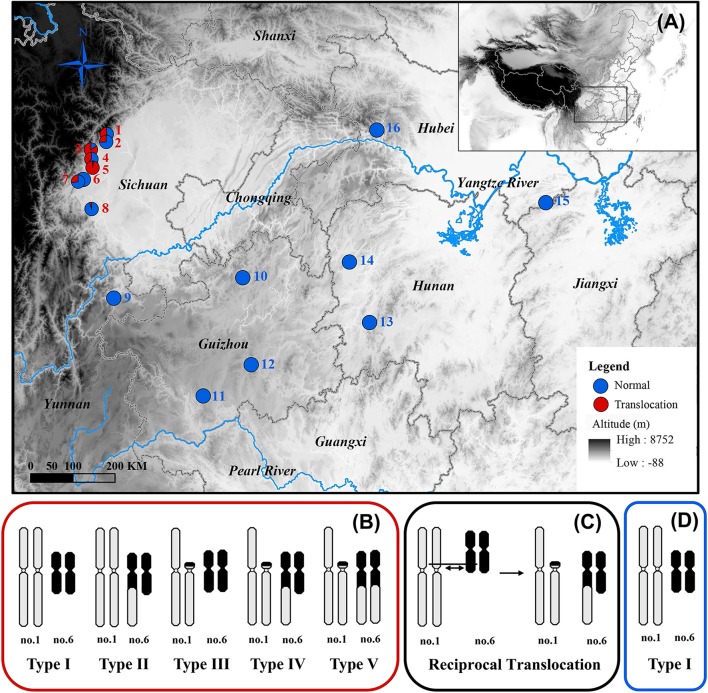
Map of sampling localities and schematic diagram of different karyotypes for *Quasipaa boulengeri*. **(A)** Sampling localities of *Q. boulengeri*. **(B)** Five different karyotypes in populations of western Sichuan Basin. **(C)** Schematic diagram of reciprocal translocation between chromosomes 1 and 6. **(D)** The only normal karyotype in populations of eastern areas. This map is created with ArcGIS (ESRI, http://www.esri.com/software/arcgis).

A fascinating case of inter-chromosomal reciprocal translocation was discovered in the spiny frog (*Quasipaa boulengeri*), which was caused by a nearly whole-arm translocation between chromosomes 1 and 6 (**Figure 1C**; Qing et al., 2012). This frog is widely distributed in the low mountainous regions along the edges of Sichuan Basin and nearby areas in southern China. The individuals with translocated karyotypes are only found in the western edges of Sichuan Basin, and inter- and intra-population karyotype variations are also found in these regions (**Figures 1A,B,D**). Additionally, the heteromorphic chromosomes are not directly related to sex, which meant both males and females share same translocated karyotypes (Types II-V) and normal karyotype (Type I) (**Figure 1B**; Qing et al., 2012). A sex-specific microsatellite marker (B08) derived from transcriptomic sequencing was identified, and it displayed perfect segregation with sex in *Q. boulengeri* of the western Sichuan Basin, revealing a possible component of genetic sex determination (GSD) and sex chromosome differentiation (Yuan S.Q. et al., 2017). Coincidentally, this area with differentiated sex chromosomes occurs in the inter- and intra-population karyotype variation. However, only one sex-linked marker without a known chromosomal location would be insufficient to discern whether translocation was the cause of sex differentiation in this area.

In the present study, we intended to develop more sex-linked markers with known chromosomal location and amplify them in wider ranges including eastern populations to investigate the role of translocation in the sex differentiation of *Q. boulengeri*. We speculated that (i) if sex-linked markers show similar sex-specific allelic distributions in eastern and western populations, translocation may have little influence on sex chromosome evolution; (ii) if sex association at sex-linked markers differs among western and eastern populations, we would analyze the relationship between translocation and the initial suppressed recombination in sex differentiation to reveal the dynamics of sex chromosome evolution.

## Materials and Methods

### Specimens and Karyotypes

A total of 687 *Q. boulengeri* adults, including 406 females and 281 males, were collected from 16 natural populations in China (**Figure 1A**) during breeding seasons from 2006 to 2016 (Qing et al., 2012; Xia et al., 2016; Yuan S.Q. et al., 2017). Populations 1–8 are all located in the western Sichuan Basin, the area where natural chromosomal translocation polymorphisms were found. Populations 9–16 were located in the southern and eastern areas of their distribution range with consistent normal chromosomal karyotype (Qing et al., 2012; **Table 1**). The phenotypic sex of sampled adults was determined according to their secondary sexual characteristics (with spiny belly in males) and from the presence of eggs (in females). Furthermore, all males and females were distinguished by gonad dissection. Mitotic metaphases were prepared, and karyotypes were identified using the same protocol as described by Qing et al. (2012). Sexes and karyotypes of all individuals are shown in **Supplementary Table S1**. Genomic DNA was extracted using the standard Proteinase K method (Sambrook and Russell, 2001). This study was carried out in accordance with the recommendations of the Animal Care and Use Committee, Chengdu Institute of Biology (CIB), Chinese Academy of Sciences (Permit Number: CIB-20121220A). The protocol was approved by the CIB Animal Care and Use Committee.

**Table 1:** Summary of sample localities, sample sizes (FN, females with normal karyotype; FT, females with translocated karyotypes; MN, males with normal karyotype; MT, males with translocated karyotypes), and F-statistics (based on six sex-linked loci; *F*_ISF_, females; *F*_ISM_, males; θ_S_, genetic diversity).

Pop no.	Pop abbr.	Locality	Longitude	Latitude	FN	FT	MN	MT	*F*_ST_	*F*_ISF_	*F*_ISM_	θ_S_
1	PZLMS	Longmenshan town, Pengzhou, Sichuan, China	103.8073	31.2438	2	14	7	1	0.24	-0.64	-0.64	4.41
2	PZCF	Cifeng town, Pengzhou, Sichuan, China	103.8001	31.1056	6	5	20	4	0.24	-0.19	-0.58	7.30
3	QCS	Mt. Qingcheng, Sichuan, China	103.3929	30.926	2	12	1	4	0.16	-0.45	-0.42	5.11
4	DYYEC	Yan’e village, Dayi, Sichuan, China	103.4399	30.7081	36	32	30	10	0.23	-0.21	-0.53	5.52
5	DYGTS	Temple Gaotang, Dayi, Sichuan, China	103.4658	30.5847	0	26	1	10	0.23	-0.79	-0.59	3.44
6	QLDZ	Daozuo township, Qionglai, Sichuan, China	103.2558	30.3167	41	0	9	0	0.18	-0.40	-0.51	4.15
7	QLTTS	Mt. Tiantai, Sichuan, China	103.1128	30.2775	21	12	18	3	0.17	-0.24	-0.44	5.77
8	EMPX	Puxing township, Emeishan, Sichuan, China	103.4675	29.6888	25	2	12	0	0.17	-0.12	-0.37	4.6 5
9	DGTX	Tianxing town, Daguan, Yunnan, China	104.0202	27.8064	28	0	21	0	0.05	0.13	-0.04	16.68
10	SYKKS	Kuankuoshui, Suiyang, Guizhou, China	107.1643	28.2331	11	0	20	0	0.05	-0.14	-0.03	7.47
11	ZYST	Shuitang town, Ziyun, Guizhou, China	106.1491	25.7158	10	0	4	0	-0.01	0.39	-0.09	5.09
12	GDYX	Yanxia township, Guiding, Guizhou, China	107.3123	26.3676	18	0	28	0	0.02	0.26	0.45	15.97
13	HJSP	Shuping township, Hongjiang, Hunan, China	110.1707	27.1766	31	0	26	0	0.08	0.30	0.15	15.35
14	BJHL	Hulu town, Baojing, Hunan, China	109.7746	28.4888	15	0	15	0	0.02	0.32	0.15	8.25
15	TSCW	Chuangwang town, Tongshan, Hubei, China	114.6774	29.4369	26	0	31	0	0.08	0.10	0.43	12.89
16	XSGQ	Gaoqiao town, Xingshan, Hubei, China	110.6109	31.2419	31	0	6	0	0.37	0.17	-0.27	6.39

### Microsatellites Isolation, Amplification, and Location

A total of 71 microsatellite loci have already been described in *Q. boulengeri* (Xia et al., 2013; Yuan et al., 2015; Yuan X.Y. et al., 2017), and we added 189 loci from transcriptomic or genomic sequencing. In total, these 260 loci were used for initial identification of sex-linkage and surveyed in four populations, including 2-PZCF, 7-QLTTS, 10-SYKKS, and 16-XSGQ (**Table 1**). After that, all sex-associated loci together with the previously mentioned sex-linked marker B08 were amplified in the above-mentioned 16 populations. Meanwhile, we also amplified five autosomal microsatellite markers, which showed no relationship with sex (Yuan S.Q. et al., 2017). The other loci with no detectable linkage with sex were ignored in the latter amplification and analyses. Primer details for each locus are listed in **Supplementary Table S2**. Each 25 μL PCR reaction volume contained 0.5 μL of undiluted genomic DNA (50–200 ng/μL), 0.5 μL of each primer (10 mM), 12.5 μL 2 × TSINGKE Blue Master Mix (Tsingke Biotech, Beijing, China), with sterile ddH_2_O as complement. The PCR protocol involved initial denaturation at 94°C for 5 min, followed by 35 cycles (94°C for 30 s, annealing temperature for 30 s and elongation at 72°C for 45 s), and a final extension at 72°C for 8 min. Negative controls were run for all amplifications. The PCR products were genotyped by Sangon Biotech Ltd., Co. (Shanghai, China). Besides, all sex-linked and five autosomal loci were mapped at the chromosome level using the same protocol as described by Yuan X.Y. et al. (2017). Briefly, we collected our target chromosomes from metaphase spreads using mechanical microdissection, and then we amplified chromosomal DNA by the single-cell whole-genome amplification technique (Sigma Chemical Co., United States). Using the chromosomal DNA as templates, we amplified microsatellite loci through PCR. If the microsatellite locus could be amplified successfully, it was considered to locate on this chromosome.

### Statistical and Cluster Analyses Based on Sex-Linked Markers

After obtaining the genotyping data for these loci, we checked allele sizes using GeneMarker v.1.95 (Softgenetics, State College, PA, United States). For sex-linked markers, allelic frequencies, expected heterozygosity (*H*_E_), F-statistics (*F*_ST_) values, and fixation indices (*F*_IS_) for males and females per population were calculated by FSTAT 2.9.3 software (Goudet, 2002). Significance levels were calculated with 5,000 randomizations and adjusted for multiple comparisons with Bonferroni corrections. To make the results look clearer, populations located in western Sichuan Basin (populations 1–8) were merged into one cluster named the western group, and the remaining populations (populations 9–16) were named the eastern group. Allelic frequencies between females and males of each locus were calculated to assess whether a difference existed between the two groups. Using the Genepop version 4.2 package (available at http://www.genepop.curtin.edu.au), linkage disequilibrium and Hardy–Weinberg exact tests were performed for each population and females with the normal karyotype of the western group.

Using six sex-linked markers, a Mantel test was employed in Arlequin 3.0 (Excoffier et al., 2005) to determine if there was significant correlation between the genetic [*F*_ST_/(1 -*F*_ST_)] and geographical distances of the localities studied. Thereafter, we performed an individual-based Bayesian clustering assignment analysis by STRUCTURE version 2.3.4 (Pritchard et al., 2000). All analyses were run in a 1,000 burn-in period and 10,000 MCMC chains with admixture and allele frequencies correlated model. We fixed *K* = 2 (ten replicates) for two groups corresponding to the number of phenotypic categories of interest (males and females). STRUCTURE analysis for females and males of the two groups were also separately performed to see whether differentiation exists within the same sexual phenotype. The graphical results were generated with the STRUCTURE PLOT program (Ramasamy et al., 2014). We also separately ran a principal component analysis (PCA) for the two groups using PCAGEN 1.2.1 software (Goudet, 1999). As PCAGEN and STRUCTURE relied on different assumptions, convergence in clustering should increase the reliability of our results. Furthermore, STRUCTURE analyses for two groups with a variety of *K* values (2–20) were also run to determine the profile of genetic clustering in the samples.

### Genetic Diversity in Western Sichuan Basin Populations

Under neutral equilibrium, population genetics theory stipulates that the amount of genetic diversity index θ was dependent on the effective population size *N*_e_, mutation rate μ, and number of chromosome copies per breeding pair c, where θ = cN_e_μ. Assuming the condition of identical effective population size and mutation rate for both sexes, the ratio of gene diversity index of autosome, X chromosome, and Y chromosome was 4: 3: 1. Given the possible male heterogamety revealed by Yuan S.Q. et al. (2017), we calculated genetic diversity for the Y chromosome (θ_Y_) and for the X chromosome (θ_X_) at each sex-linked locus, as well as for autosomes (θ_A_) separately at each autosomal locus per population in the western Sichuan Basin. Genetic diversity index θ was calculated from expected heterozygosity [equation: θ = (1/(1-H_E_)^2^-1)/2] assuming a stepwise mutation model (Kimura and Ohta, 1975). Thereafter, the expected consensus values, [*E*(θ_Y_) and *E*(θ_X_)], were calculated assuming the aforementioned conditions, as *E*(θ_Y_) = (θ_Y_ + θ_X_)/4 and *E* (θ_X_) = 3(θ_Y_ + θ_X_)/4. To make the results concise and clear, an average θ of these loci for each population was used in the western group.

### Phylogenetic Inference

To identify whether the reciprocal translocation evolved in *Q. boulengeri* was ancestral or a condition that occurred thereafter, two fragments from the mitochondrial genome were selected for phylogenetic analysis. A ∼628 bp fragment of *cox1* was amplified in 62 specimens using the primers VF1d and VR1d from Ivanova et al. (2006). The second fragment is part of the *cytb* gene and 837 bp in length. Together with sequences from previous study (Qing et al., 2012; Xia et al., 2016), a total of 212 specimens were used for molecular phylogenetic analysis. Detailed sequence information is shown in **Supplementary Table S1**. Sequence alignment and haplotype identification were conducted with MEGA7.0 (Kumar et al., 2016) and DnaSP v.6.10 (Rozas et al., 2017). Two species closely related to *Q. boulengeri* were chosen as outgroup taxa, *Q. robertingeri* and *Q. spinosa*. The best-fit model was estimated using the Bayesian information criterion (BIC) implemented in PartitionFinder v2.1.1 (Lanfear et al., 2017). We ran four Markov chains for 10 million generations, retaining every 1000th sample from posterior distribution, and starting values for each chain were chosen randomly in MrBayes 3.2 (Ronquist et al., 2012).

## Results

### Sex-Linked Loci and Sex Chromosomes in *Q. boulengeri*

**FIGURE 2:**
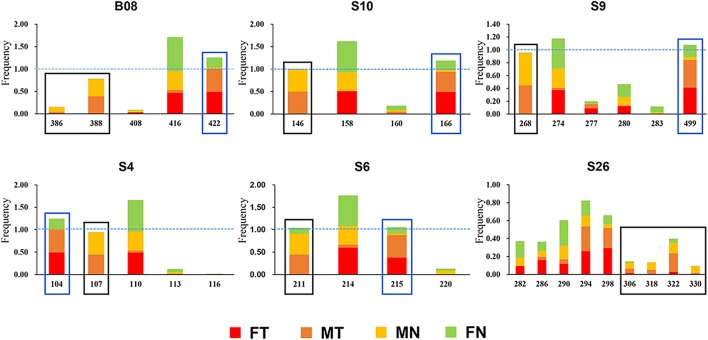
Allelic frequencies of six sex-linked loci in western Sichuan Basin. FN, females with normal karyotype; FT, females with translocated karyotypes; MN, males with normal karyotype; MT, males with translocated karyotypes. Black box, male-specific alleles; blue box, translocation-specific alleles.

The karyotype results were consistent with Qing et al. (2012). All specimens have a diploid number of 2n = 26 chromosomes, and inter- and intra-population karyotype variations were found only in western Sichuan Basin. Five different karyotypes (Types I–V, **Figure 1B**) were observed. Type I and Type IV, which were more common, resulted from reciprocal translocation occurring between chromosomes 1 and 6 (**Figure 1C**). Among the 260 screened microsatellite loci, five markers (S4, S6, S9, S10, and S26) showed evidence of sex-linkage. Overall, together with B08, all six sex-linked loci showed male-specific alleles and revealed sex-specific allelic distributions in the western Sichuan Basin (**Figure 2**, Black box), indicating male heterogamety (XY systems) and sex differentiation.

The results of microsatellite mapping showed that five sex-linked loci (S4, S6, S10, S26, and B08) were assigned to chromosome 1, and reciprocal translocation occurred on this chromosome. It was concluded that locus S9 was also located on chromosome 1, since the translocated version of locus S9 harbored a null allele in most individuals with translocated karyotypes. Such amplification may stem from either a mutation in the priming site or a deletion of the translocated chromosome-linked microsatellite, so we used a size value (499) to represent this null allele in the following analyses (**Supplementary Table S1**). All five autosomal loci failed to amplify in the DNA of chromosome 1 sets. Linkage disequilibrium tests of overall translocated and normal karyotypic individuals showed that the six sex-linked markers were tightly linked. It gave strong support that chromosome 1 was sex-linked and GSD existed in the western Sichuan Basin.

### Geographic Variation of Sex-Linked Loci

**FIGURE 3:**
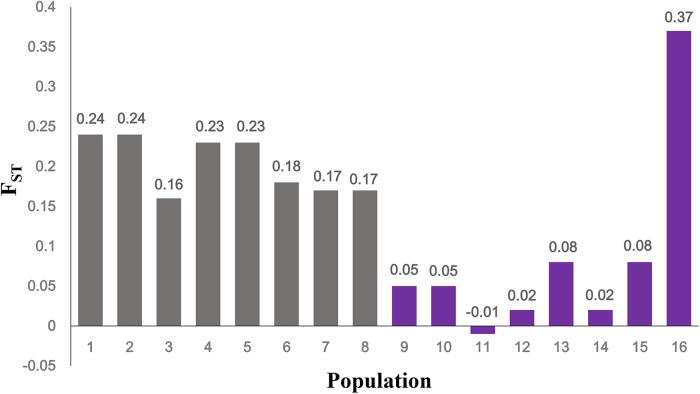
*F*_ST_ between females and males at sex-linked loci. Gray, populations in western Sichuan Basin; purple, populations in eastern areas.

In the western Sichuan Basin, for loci S4, S9, S10, and B08, 125 of 130 males displayed male-specific alleles, which were absent from nearly all females (only two exceptions out of 236 females) and the remaining five males. Interestingly, the remaining five males were all single-testis frogs, and the two exceptional females were ovary-degenerated frogs, implying that sex reversal may exist in a few individuals of *Q. boulengeri*. Locus S6 displayed good segregation with sex in populations 1–5 but not in populations 6–8. Similarly, locus S26 was sex-linked in populations 1–3 and 5, but it demonstrated no relationship with sex in populations 4 and 6–8. The *F*_ST_ values between sexes were greater than 0.15 per population in the western Sichuan Basin (**Table 1** and **Figure 3**), indicating that strong and significant differentiation did exist between sexes. Both females and males had strong heterozygosity excess (- 0.79 < *F*_ISF_ < - 0.12 and – 0.374 < *F*_ISM_ < - 0.639, respectively, **Table 1**).

Comparatively, in populations 9–16 of the southern and eastern distribution area (eastern group), we did not observe any difference in allelic distributions between males and females, both in all six sex-linked markers (**Supplementary Figure S1**) and other verified loci. Accordingly, based on six markers sex-linked in the west, the *F*_ST_ values between sexes were very low, and the *F*_IS_ values were larger than those of populations 1-8 both for males and females with an exception in population 16-XSGQ (**Table 1** and **Figure 3**). In addition, the results of linkage disequilibrium tests for this group showed a slight but variable linkage disequilibrium with different populations showing different linkage between loci. The Mantel test revealed significant correlation between geographical distances and genetic distances for all pairs of populations (*r* = 0.583, *p* < 0.001).

### Cluster Analyses Based on Sex-Linked Markers

**FIGURE 4:**
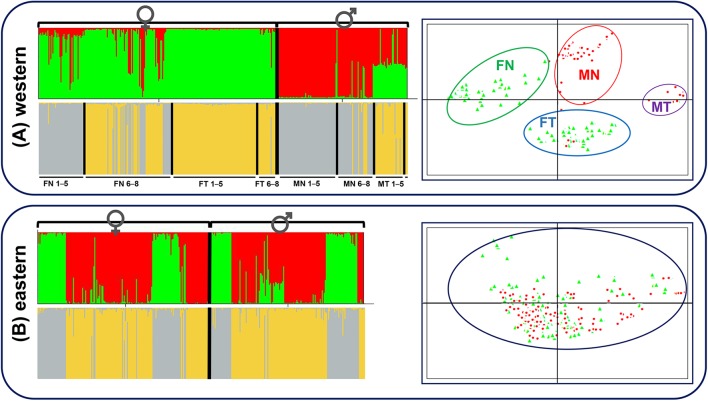
STRUCTURE and PCAGEN plots for six sex-linked markers in western and eastern groups. Triangle, females; circle, males. **(A)** Western group, individuals were assigned to the respective clusters mainly according to sexes and karyotypes. FN, females with normal karyotype; FT, females with translocated karyotypes; MN, males with normal karyotype; MT, males with translocated karyotypes. 1–5, individuals from populations 1–5; 6–8, individuals from populations 6–8. MT 6–8 only included three individuals and was not labeled. **(B)** Eastern group, assignments of females and males to the green and red clusters are independent of sex.

Both STRUCTURE and PCA analyses gave accordant results that distinct patterns existed not only for sexual phenotype but also for translocated karyotype at sex-linked makers in the western group. Except for seven possibly sex-reversed frogs, all individuals were clustered mainly into two groups according to sexual phenotype, females into green clusters and males into red clusters (*K* = 2, **Figure 4A**). When considering females separately, the frogs were clustered into two groups mainly according to karyotypes (*K* = 2, **Figure 4A**). The vast majority of females with translocated karyotypes (FT) were divided into yellow clusters (FT 1–5 and FT 6–8), whereas females with normal karyotype (FN) were divided into two clusters, individuals from populations 1–5 belonged to gray clusters (FN 1–5) and individuals from populations 6–8 belonged to either gray or yellow clusters (FN 6–8). The males presented the same situation, where translocated (MT) and normal (MN) karyotypic individuals were clustered into two groups, with several normal karyotypic individuals from populations 6–8 belonging to yellow clusters (MN 6–8). PCAGEN showed consistent results, individuals of the western group were clustered into four groups, mainly based on their phenotypic sexes and karyotypes as follows: (i) green clusters included all normal karyotypic females (FN), (ii) red clusters were normal karyotypic males (MN), (iii) blue clusters were females with translocated karyotypes (FT), and (iv) purple clusters were males with translocated karyotypes (MT). Seven possible sex-reversed individuals and several normal karyotypic individuals in populations 6–8 were still clustered into matched groups (**Figure 4A**).

Contrastingly, STRUCTURE analysis of the eastern group showed that individuals were clustered into two groups without relationship to their sex (*K* = 2, **Figure 4B**). The separate cluster analyses of females and males were also consistent with the overall analysis without a hidden genetic structure related to phenotypic sexes (**Figure 4B**). Irrespective of the *K* value, there was no obvious link between sex and clusters. PCA analysis demonstrated that males and females appeared to be randomly distributed within one single cluster (**Figure 4B**).

STRUCTURE analyses with a variety of *K* values performed over two groups were provided in **Supplementary Figure S2**. The maximum of ΔK was found for *K* = 3 in the western group. Individual assignments almost matched the sexual phenotypes and karyotypes, females with normal karyotype belonged to the green cluster, females with translocated karyotypes and some normal karyotypic individuals from populations 6-8 belonged to the blue cluster, males with normal karyotype belonged to the red cluster, and males with translocated karyotypes belonged to the half-red-half-blue cluster (**Supplementary Figure S2**). The maximum of ΔK was found for *K* = 2 in the eastern group, individual assignments were identical with **Figure 4B**, as phenotypic sex had nothing to do with the clusters. STRUCTURE analyses performed for each population are also provided in **Supplementary Figure S2** (*K* = 2). Populations in the western group were clustered into two groups, mainly according to sexual phenotype, with an exception in population 2-PZCF. Furthermore, assignments to population 2-PZCF matched the karyotypes of individuals, all translocated karyotypic frogs belonging to the green cluster and normal karyotypic individuals belonging to the red cluster, indicating that reciprocal translocation affecting genetic structure was stronger than sex-linked differentiation in this population (**Supplementary Figure S2**). However, for populations in the eastern group, STRUCTURE analyses showed that phenotypic sex had nothing to do with the clusters (**Supplementary Figure S2**).

### Genetic Diversity Polymorphisms in Western Sichuan Basin

In all amplified populations, five autosomal markers displayed no differences of allelic distribution between males and females, which was consistent with a previous study (Yuan S.Q. et al., 2017). In the western Sichuan Basin, the average expected heterozygosity (H_EA_) of each population ranged from 0.57–0.74 and average genetic diversity (θ_A_) ranged from 3.64–37.51 (**Table 2**). No significant linkage disequilibrium or deviation from Hardy–Weinberg equilibrium was detected within populations at five autosomal markers.

**Table 2:** Genetic diversity (θ) for populations in western Sichuan Basin.

Pop no.	Pop abbr.	θ_Y_	θ_X_	θ_A_	E(θ_Y_)	E(θ_X_)	E(θ_A_)	θ_XT_	θ_XN_
1	PZLMS	0.21	1.96	4.38	0.54	1.63	2.17	0.00	0.41
2	PZCF	0.35	2.74	18.87	0.77	2.31	3.08	0.00	1.53
3	QCS	0.19	2.35	37.51	0.64	1.91	2.54	0.13	2.18
4	DYYEC	0.00	1.48	17.13	0.37	1.11	1.48	0.25	0.31
5	DYGTS	0.05	1.66	3.64	0.43	1.28	1.71	0.02	0.90
6	QLDZ	0.06	2.11	6.22	0.54	1.63	2.17	–	–
7	QLTTS	0.13	2.79	21.27	0.73	2.19	2.92	–	–
8	EMPX	0.00	1.94	22.52	0.49	1.46	1.94	–	–
Average		0.12	2.13	16.44	0.56	1.69	2.25	0.08	1.07

*θ_Y_ for Y chromosome, θ_X_ for X chromosome, θ_A_ for autosome; E(θ), expected genetic diversity; θ_XT_ for translocated X chromosome and θ_XN_ for normal X chromosome*.

Owing to the absence of male recombination and strong sex differences in allelic frequencies, the X and Y haplotypes were phased in males and females for sex-linked markers using the private male alleles. Based on sex-linked markers displaying perfect segregation with sex, we computed the heterozygosity (H_E_) and genetic diversity (θ) both for Y and X chromosomes separately (**Supplementary Table S3**). Compared with the expected consensus values under neutral equilibrium, [*E*(θ_Y_) and *E*(θ_X_)], sex-linked loci generally showed a deficit of diversity on Y in each population with a few exceptions (**Supplementary Table S3**). An average value was used to reflect the overall genetic diversity for each population. In each population, θ_X_ was 7.83–∞ times greater than θ_Y_ (**Table 2**). This finding suggested that the difference in chromosome copy number cannot account for the observed reduction in diversity on the Y chromosome. In addition, autosomal genetic diversity (θ_A_) was 2.19–15.94 times greater than θ_X_, significantly higher than the expected ratio (4:3) at neutral equilibrium, indicating an extreme heterozygosity deficit on the X chromosomes and excessive autosomal heterozygosity (**Table 2**). In other words, the genetic diversities of both X and Y chromosomes were reduced.

As mentioned above, all six sex-linked markers were located on chromosome 1 and related to reciprocal translocation. Translocated karyotypic individuals, including females and males, had translocation-specific alleles at each locus, which were labeled by a blue box in **Figure 2**. Although locus S6 was a microsatellite locus of triplet repeats, all translocated karyotypic individuals displayed the same allele with a single nucleotide insertion, changing the translocated allele size to 215. In addition, locus S26 displayed different translocation-specific alleles in different populations; therefore, it was not labeled in **Figure 2**. As both MT and FT shared same specific alleles and belonged to X haplotypes, it appeared that translocation was related to sex chromosomes, and the translocated version of chromosome 1 was the X chromosome. Accordingly, the sex chromosomes of four kinds of individuals could be defined as follows: (i) the translocated karyotypic males consisted of a Y and a translocated X chromosome, (ii) the translocated karyotypic females consisted of a normal X and a translocated X chromosome, (iii) the normal karyotypic males consisted of a Y and a normal X chromosome, and (iv) the normal karyotypic females consisted of a pair of normal X chromosomes. Based on well-separated specific alleles in translocated individuals, we computed the genetic diversity for translocated X chromosomes (θ_XT_) and for the remaining normal X chromosomes (θ_XN_). In all populations, the θ_XT_ values were 0 or close to 0, and the θ_XN_ values were all greater than θ_XT_ (**Table 2**). This meant that the genetic diversity of the translocated X chromosome was very low, and recombination was almost entirely halted between the translocated and the normal chromosomes.

**FIGURE 5:**
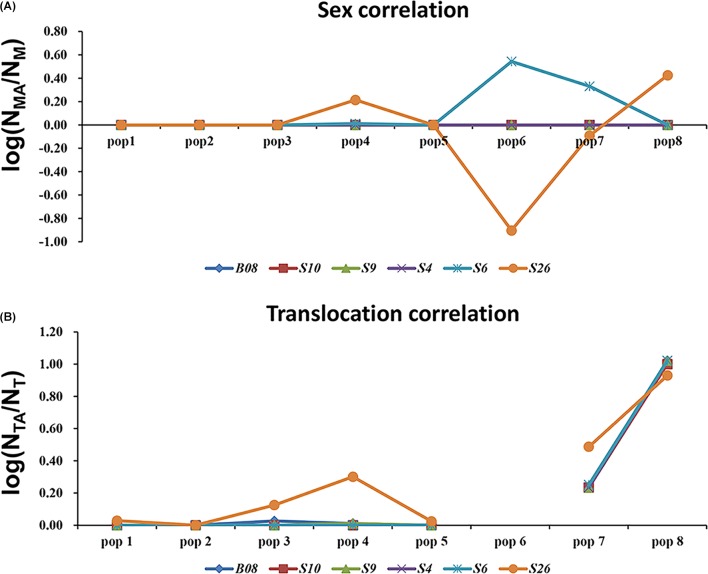
Analyses of sex-association and translocation-linkage for sex-linked loci in populations of western Sichuan Basin. **(A)** Sex correlation, N_MA_, the number of male-specific alleles; N_M_, the number of males. **(B)** Translocation correlation, N_TA_, the number of translocation-specific alleles; N_T_, the number of translocated karyotypic individuals. The value should be 0 in the case of recombination suppression, deviation from this value would point to unrestricted recombination. Population 6-QLDZ did not include any individuals with translocated karyotype, hence there was a break in the line with no values shown for population six.

Even in the loci that displayed both sex association and translocation linkage in the western Sichuan Basin, we found that the relevance varied in different populations. The correlation coefficients were calculated using the number of males and male-specific alleles, as well as translocated individuals and translocation-specific alleles (**Figure 5**). Notably, some loci did not show good segregation with sex and karyotypes in several populations. In these cases, male-specific and translocation-specific alleles were still assumed to be the same as in the populations with perfect sex-linkage and karyotype-correlation, which were labeled by black and blue boxes in **Figure 2**. For sex-association, sex chromosome differentiation and recombination was totally suppressed at four loci (S4, S9, S10, and B08) in each population. However, the other two loci (S6 and S26) varied in different populations. Locus S6 was sex-linked in all individuals of populations 1–5 but demonstrated no relationship with sex in normal males of populations 6–8. Locus S26 showed a similar pattern to S6; it was sex-linked in all individuals of populations 1–3 and 5, but it demonstrated no relationship with sex in males with normal karyotype of populations 4 and 6–8 (**Figure 5A** and **Supplementary Table S1**). For translocation-linkage, five loci (S4, S6, S9, S10, and B08) showed good segregation with karyotypes in populations 1–5, suggesting the existence of suppressed recombination caused by translocation. However, all six loci showed no detectable linkage with karyotypes in populations 6–8 (**Figure 5B** and **Supplementary Table S1**), indicating unlimited recombination. It was important to note that six loci were sex-linked and showed male-specific alleles in translocated karyotypic males of populations 6–8. This pattern means that suppressed recombination of sex differentiation existed in all populations of the western Sichuan Basin. Comparatively, suppressed recombination caused by translocation only existed in populations 1–5.

Given the sex-specific and translocation-specific distribution of alleles, we test linkage disequilibrium for six sex-linked markers in all normal karyotypic females. The results showed that linkage was no longer present at all loci; only some of them were partially linked, and the other loci could recombine normally (**Supplementary Table S4**). Based on this, a simplified drawing was made to show the relative position of the six sex-linked markers (**Supplementary Figure S3**).

### Phylogenetic Analysis

**FIGURE 6:**
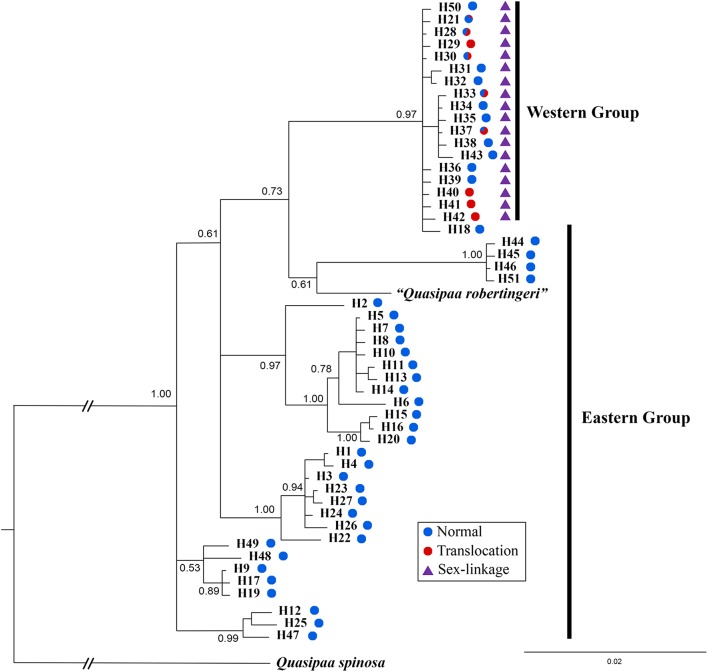
Bayesian phylogenetic reconstruction using *cox1* and *cytb* sequence data. The number at each node is the Bayesian posterior probability supporting the phylogram displayed.

The *cox1* + *cytb* data set had 51 haplotypes and 1463 nucleotide sites (**Supplementary Table S1**). The 50% majority consensus tree was illustrated in **Figure 6**. All *Q. boulengeri* samples formed a highly supported clade with *Q. robertingeri*, which was resolved as a synonym of the former (Che et al., 2009). Within *Q. boulengeri*, specimens possessing translocated and normal karyotypes from the western group clustered into a strongly supported clade (**Figure 6**). Individuals from the eastern group appeared on different clades. Besides, no matter whether individuals had normal or translocated karyotypes, a distinct divergence among sexual phenotypes at sex-linked loci were exhibited by the western group (**Figure 6**, triangle). Combined with the sex-association and translocation-linkage distributions (**Figure 5**), it was revealed that the establishment of the sex-linkage might evolve independently of reciprocal translocation, and reciprocal translocation might not be the initial causative mechanism of suppressed recombination for sex differentiation in this frog.

## Discussion

### Geographic Variation of Sex Chromosome Differentiation

In populations of the western Sichuan Basin, the sex specificity of allelic distributions, the highly negative *F*_IS_ value, the high *F*_ST_ value between males and females, and the cluster analyses all showed that these six sex-linked loci were associated with sex and lay on the non-recombining region of sex chromosomes. Overall, the X and Y chromosomes of *Q. boulengeri* were differentiated to different degrees and could not recombine freely, leading to stable GSD. In contrast, we did not observe any signals of sex linkage in populations of the eastern group. Lack of allelic frequency differences, the relatively low *F*_ST_ values between sexes, and the chaotic clusters all suggested sex differentiation did not exist at the markers we studied, indicating uncertain sex determination mechanisms in the eastern group. Clearly, our results presented a striking geographic variation in the differentiation of sex chromosomes and sex determination based on sex-linked markers.

In amphibians, most of the species investigated so far present a genetic component to sex determination using various methods (Eggert, 2004). However, the instability and diversity of sex differentiation are not rare. In *Rana japonica*, some loci were found to be definitively linked with the sex-determining genes in some populations, whereas this was not the case in other populations (Sumida and Nishioka, 1994). In *Rana temporaria*, Rodrigues et al. (2014) found that sex differentiation at the linkage group 2 was strongest in the northern boreal population but null in the southernmost population, indicating decreasing levels of sex linkage with decreasing latitude across Sweden. Recently, the sex-linked markers developed in the North American green frog (*Rana clamitans*) were predominantly robust to geographic variation at a scale of up to 52 km among source ponds, and among these markers, four SNP and four PA (presence/absence of restriction fragments) loci showed no sex linkage at the northernmost pond, yet they were tightly linked to sex at other ponds (Lambert et al., 2016).

### Suppressed Recombination of Sex Chromosomes Evolved Independently of Reciprocal Translocation in *Q. boulengeri*

Populations in the western Sichuan Basin harbored stable GSD and variable differentiated sex chromosomes. Lack of recombination between sex chromosomes existed in all males of the western group, regardless of karyotype variations. The suppressed recombination between sex chromosomes may have occurred differently in this region, which can explain why the markers S6 and S26 seemed not to be sex-linked in males with normal karyotype of western populations 6–8, because S6 and S26 may lie on distal segments of non-recombining regions (**Supplementary Figure S3**). Males with translocated karyotypes showed male-specific alleles at all six sex-linked loci, whereas normal karyotypic males displayed two kinds of sex chromosome differentiation (six vs. four sex-linked loci). The different levels of differentiation might have resulted from the translocation. However, in eastern populations, both similar allelic frequency distributions between sexes and chaotic clusters suggested unrestricted recombination at the markers we studied.

Our results agree with the assumption that sex association at sex-linked markers differs among the western and eastern populations, and such differences seem to be coincidental with translocation. However, genetic differentiation between sexes in the western populations was not directly correlated with karyotypes, as it was found in individuals with both normal and translocated karyotypes. There are two scenarios to explain the occurrence of suppressed recombination in sex chromosome evolution. One plausible explanation was that translocation was the trigger to acquire sex-linked loci and was the initial causative mechanism of suppressed recombination for sex differentiation. In this hypothesis, sex-linked loci should relate to recombination suppression caused by translocation or karyotype variations. Moreover, sex-linked loci should show translocation-linkage in all populations with translocated karyotypic individuals. However, such hypothesis was refuted by our results, male-specific and translocation-specific allelic frequency distributions suggested that recombination of sex-differentiation ceased in populations 1–8, but recombination suppression caused by translocation did not exist in populations 6–8 (**Figures 2**, **4**, **5**).

An alternative explanation was that sex differentiation evolved independently of translocation, and translocation may not be the root cause of initial suppressed recombination. In this case, sex-linked loci may be associated with translocation or completely unrelated, which can explain the different levels of ceased recombination caused by translocation in different populations (**Figures 2**, **4**, **5**). This explanation was also supported by both Mantel test and phylogenetic analysis (**Figure 6**). Significant correlation between geographical and genetic distances meant that geographic distance may play an important role in geographical variation of sex chromosome differentiation in this spiny frog. Phylogenetic inference showed all individuals processing heteromorphic and homomorphic karyotypes from the western Sichuan Basin, and several individuals from eastern regions belonged to a strongly supported clade, which was consistent with Qing et al. (2012). As individuals of the eastern group appeared on different clades, it is reasonable to assume that the eastern group would be an ancestral condition. It indicated that *Q. boulengeri* originated in the east, and the western group was derived. This was consistent with previous study, where Yan et al. (2013) found that the unification of the upper and middle Yangtze River in the Three Gorges mountain region (eastern group) mediated downstream colonization of this frog. Several of the same haplotypes and sexual phenotypes were shared by individuals with normal and translocated karyotypes (**Figure 6**). Besides, suppressed recombination of the sex-linked markers has been observed in all individuals of the western group (**Figures 2**, **4**–**6**). That means suppressed recombination of the sex-linked markers exists without translocation, especially for normal males. Similar to most species, such type of suppressed recombination may be caused by the acquisition of sex-determining loci and/or sexually antagonistic mutations (Charlesworth et al., 2005; Kirkpatrick, 2017). Based on this evidence, it was highly possible that suppressed recombination for sex chromosome differentiation appeared independently of translocation in the western Sichuan Basin. In other words, translocation could not be the root cause of sex differentiation in *Q. boulengeri*.

CRs were not the root causes of sex differentiation, which was also supported by some reports from other species. Bergero et al. (2008) concluded that Y chromosome of *Silene latifolia* has been derived through multiple rearrangements; however, none of the rearrangements so far detected were involved in stopping X–Y recombination. In three spine sticklebacks (*Gasterosteus aculeatus*), a neo-sex chromosome system has recently evolved due to a fusion between the Y chromosome and an autosome in the Japan Sea lineage. Chromosome-wide differentiation patterns and phylogenetic inferences indicated that the ancestral sex chromosomes were extensively differentiated before the divergence of the Japan Sea lineage (Natri et al., 2013). In *Neurospora tetrasperma*, a fungus that lacks recombination over most of its largest chromosome, genomic data sets surprisingly reveal that inversions of the non-recombining regions were derived and not the cause of the recombination suppression (Sun et al., 2017). Empirical investigations provided evidence that repetitive sequences could accumulate on regions of suppressed recombination and promote CRs (Charlesworth et al., 1994; Charlesworth, 2017). In addition, the hypothesis of relaxed selection holds that once recombination has been halted in the heterogametic sex, selection to maintain gene order is abolished, and selection against CRs would be relaxed and potentially increase their frequency in a population (Flot et al., 2013; Knief et al., 2016; Sun et al., 2017). In other words, CRs may be the consequence of suppressed recombination, rather than just the cause. However, the previous studies of CRs mostly concentrated on inversions or fusions (Wilson and Makova, 2009; Natri et al., 2013). Reciprocal translocation had rarely been studied, as stable intra-population polymorphism for a reciprocal translocation is quite rare among sexually reproducing animals. Our results suggested that reciprocal translocation may also be less likely to be selected in regions of suppressed recombination, similar to inversions and fusions.

### Translocation May Accelerate Sex Chromosome Divergence in *Q. boulengeri*

For sexual diploid organisms, lack of recombination caused by sex chromosomes differentiation alone would give the expected 3:1 ratio of X to Y diversity. In our study, comparison of sex-linked and autosomal microsatellites of *Q. boulengeri* revealed that the genetic diversity of both Y and X chromosomes was greatly reduced. This reduction cannot be accounted for by merely the number of chromosome copies per breeding pair. There were two possible causes for the lower-than-expected level of polymorphism: sex-linked microsatellites located on the sex chromosome might show a decreased mutation rate (μ), or the effective population size (*N*_e_) of the sex chromosome was reduced below simple expectation. Very little evidence was available about the microsatellite mutation rates in amphibians, while Kayser et al. (2000) concluded that mutation rates of human Y-chromosomal microsatellites are consistent with autosomal microsatellites. Hence, we assumed the mutation rates of microsatellites located on the non-recombining region of the sex chromosomes to be consistent with autosomal loci in *Q. boulengeri*, similar to humans. Therefore for *Q. boulengeri*, reduced genetic diversity is very likely to be a consequence of reduced N_e_ of both the Y and X chromosomes.

The reduction of *N*_e_ of sex chromosomes would accelerate the fixation of mildly deleterious mutations by random drift and reduce variability at linked sites (Charlesworth, 1994; Hudson and Kaplan, 1995). Generally, CRs are deleterious when heterozygous because of suppressed recombination but have normal fitness when homozygous (White, 1978). When rearrangements occurred on sex chromosomes, the crossing over between sex chromosomes would be suppressed, making sex chromosomes vulnerable to the operation of forces that lead to a reduction in their effective population size and thus enhancing the power of drift (Charlesworth, 2002; Kirkpatrick, 2010). A side effect of repressed recombination on Y or W chromosomes is that natural selection is inefficient; thus, fixation can occur in a local area through random genetic drift (Lande, 1979; Bachtrog, 2013). Besides, selection on linked loci (background selection and selective sweeps) might combine with drift to drastically accelerate the rate of fixation of deleterious mutations and the loss of neutral diversity (Charlesworth et al., 1993; Slatkin, 1995). In *Q. boulengeri*, genetic diversity of translocated sex chromosomes was very low (**Table 2**), meaning that reciprocal translocation inhibited recombination and reduced genetic diversity of sex chromosomes in individuals with rearranged karyotypes.

Besides, the proportions of translocated individuals were variable in different populations, and the different levels of sex chromosome differentiation might be consequences of translocation in the western Sichuan Basin. From limited sampling data, we found there were relatively high-proportioned individuals with translocated karyotypes in populations 1–5 but low in populations 6–8. The sex chromosomes of normal males were more differentiated in populations 1–5 than in populations 6–8, indicating that translocation promoted sex chromosome divergence. Therefore, it is possible that after the appearance of initial recombination suppression of sex chromosomes, reciprocal translocations might expand the regions of recombination suppression and reduce the N_e_ of sex chromosomes, furthering which the genes associated with sex were fixed mainly by enhanced drift, background selection, and selective sweeps, facilitating sex chromosome differentiation and stabilizing sex determination mechanism.

In short, the initial cause of sex differentiation in *Q. boulengeri* may be something other (e.g., sexually antagonistic selection) than translocation (Charlesworth et al., 2005; Kirkpatrick, 2017), and that was why sex-linkage existed in all individuals of the western group, regardless of karyotype. Our results offered empirical evidence to reveal the role of reciprocal translocation in sex chromosome evolution. Although translocation was not the ultimate cause of sex differentiation, it may accelerate sex chromosome divergence by reducing the genetic diversity and effective population size of sex chromosomes.

## Data Archiving

The datasets supporting this article have been uploaded as part of the **Supplementary Material**. The sequences of frogs in this study were deposited in GenBank with accession numbers MH029835–MH029854 and MH428058–MH428099.

## Author Contributions

YX and XZ designed the study. XY performed the experiments. XY and YX analyzed the data and wrote the paper. XZ improved the paper.

## Conflict of Interest Statement

The authors declare that the research was conducted in the absence of any commercial or financial relationships that could be construed as a potential conflict of interest.

## Supplementary Material

FIGURE S1 Allelic frequencies of six loci sex-linked at west in eastern populations. Female: white bars; male: gray bars.

FIGURE S2 STRUCTURE analyses for two groups and per population.

FIGURE S3 Relative position of sex-linked loci.

TABLE S1 Populations, sexes, karyotypes, genotypes, *cox1* and *cytb* sequences of all individuals. Seven individuals with possible sex reversal are highlighted (in red font). F, female; M, male.

TABLE S2 Primer details for microsatellite loci.

TABLE S3 Genetic diversity of sex-linked loci per population in western Sichuan Basin. H_EY_, expected heterozygosity for Y chromosome; H_EX_, expected heterozygosity for X chromosome; θ_Y_, genetic diversity index of Y chromosome; θ_X_, genetic diversity index of X chromosome; E(θ_Y_), expected genetic diversity index of Y chromosome; E(θ_X_), expected genetic diversity index of X chromosome.

TABLE S4 *P*-values for pairwise linkage disequilibrium analyses among sex-linked loci.
