# Supplementary figures and images for: Suppressed Recombination of Sex Chromosomes Is Not Caused by Chromosomal Reciprocal Translocation in Spiny Frog (Quasipaa boulengeri)
Source: Front Genet. 2018 Aug 27;9:288. doi: 10.3389/fgene.2018.00288 (PMC6119705; doi:10.3389/fgene.2018.00288)

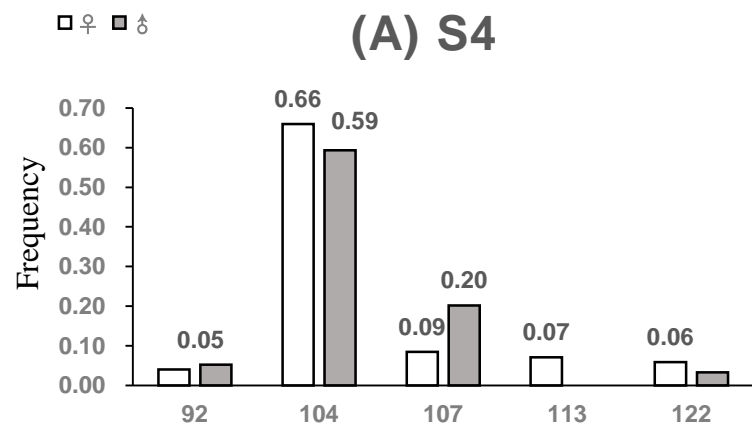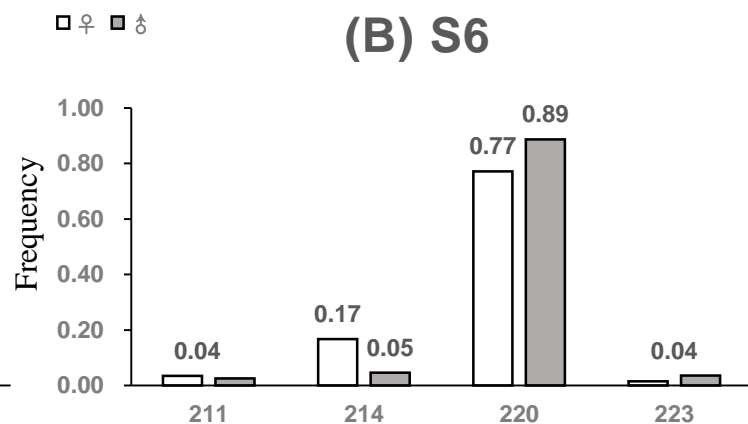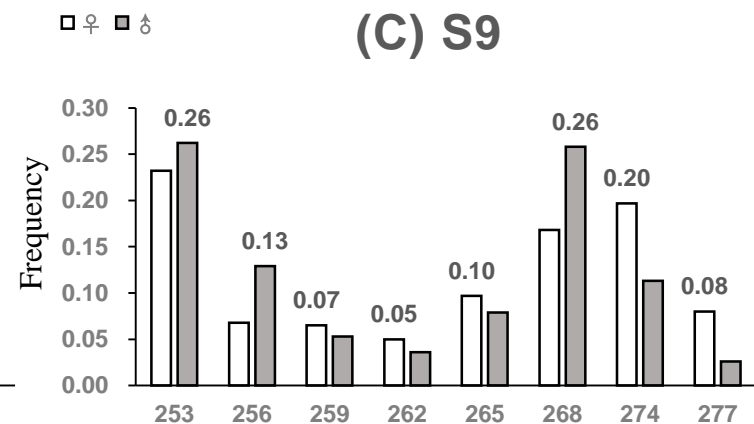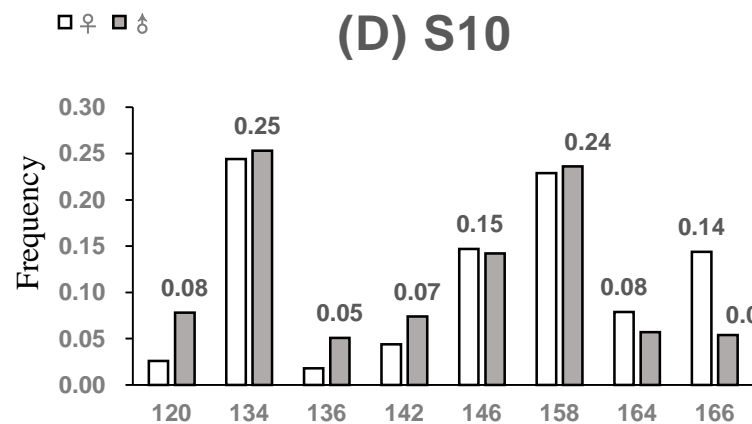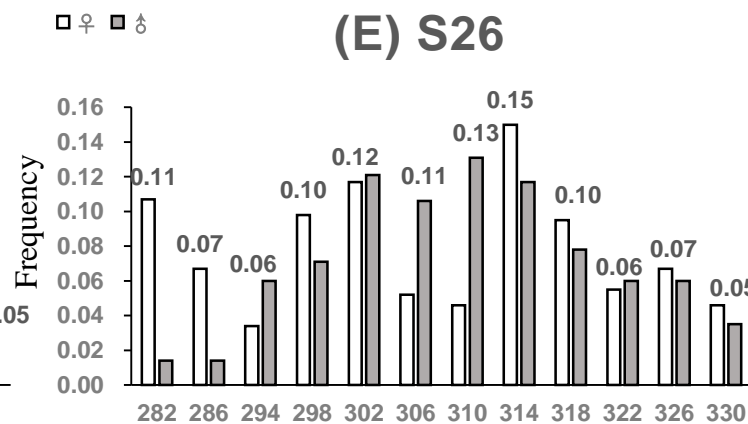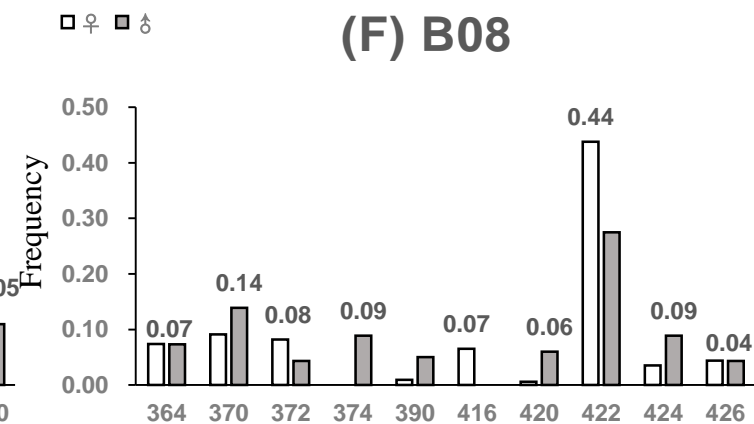

Supplement: FIGURE S1 — Allelic frequencies of six loci sex-linked at west in eastern populations. Female: white bars; male: gray bars. [file Image_1.PDF]

## (A) Western group

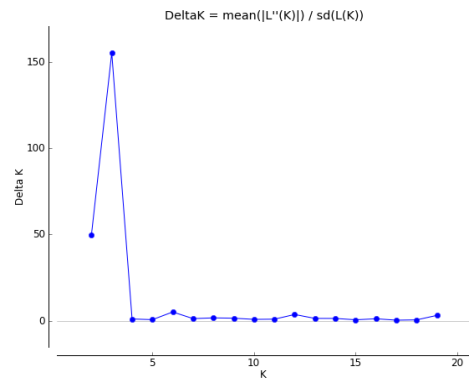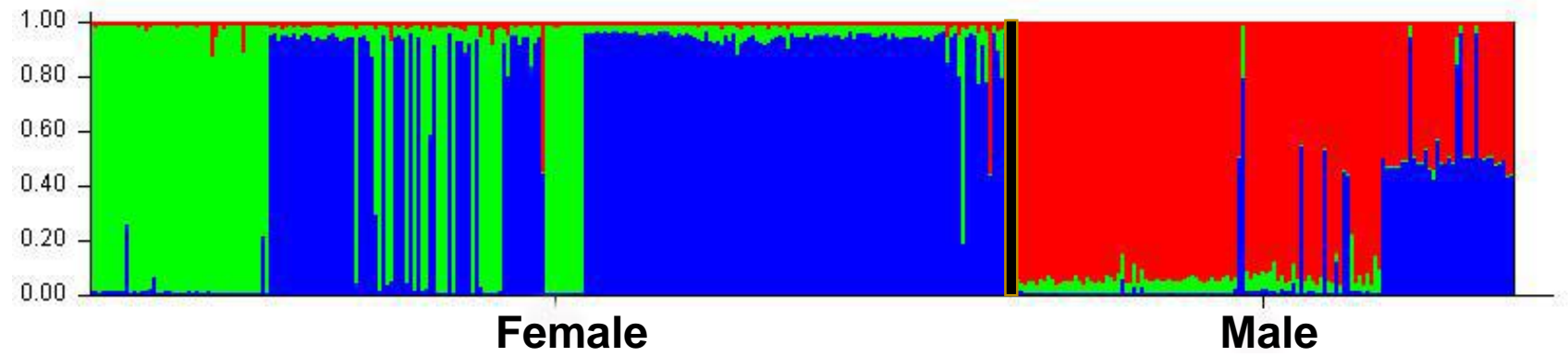

## (B) Eastern group

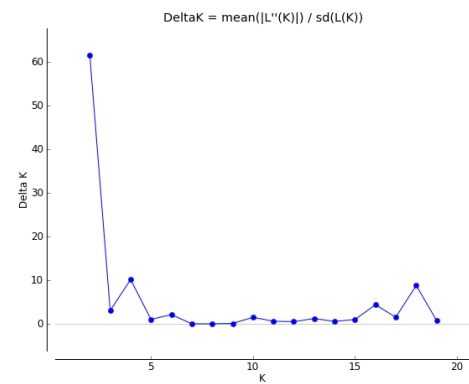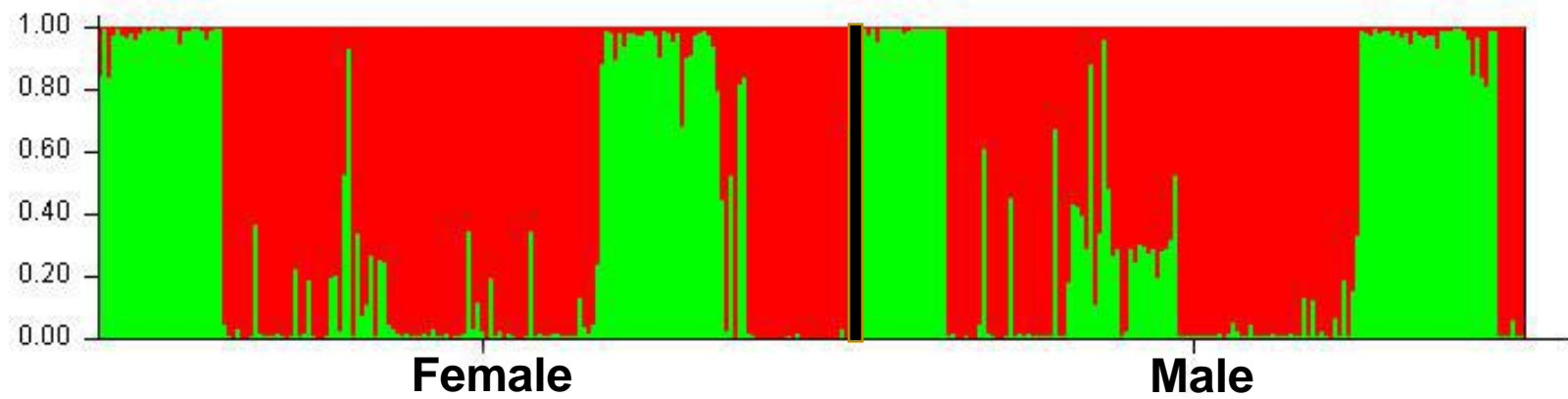

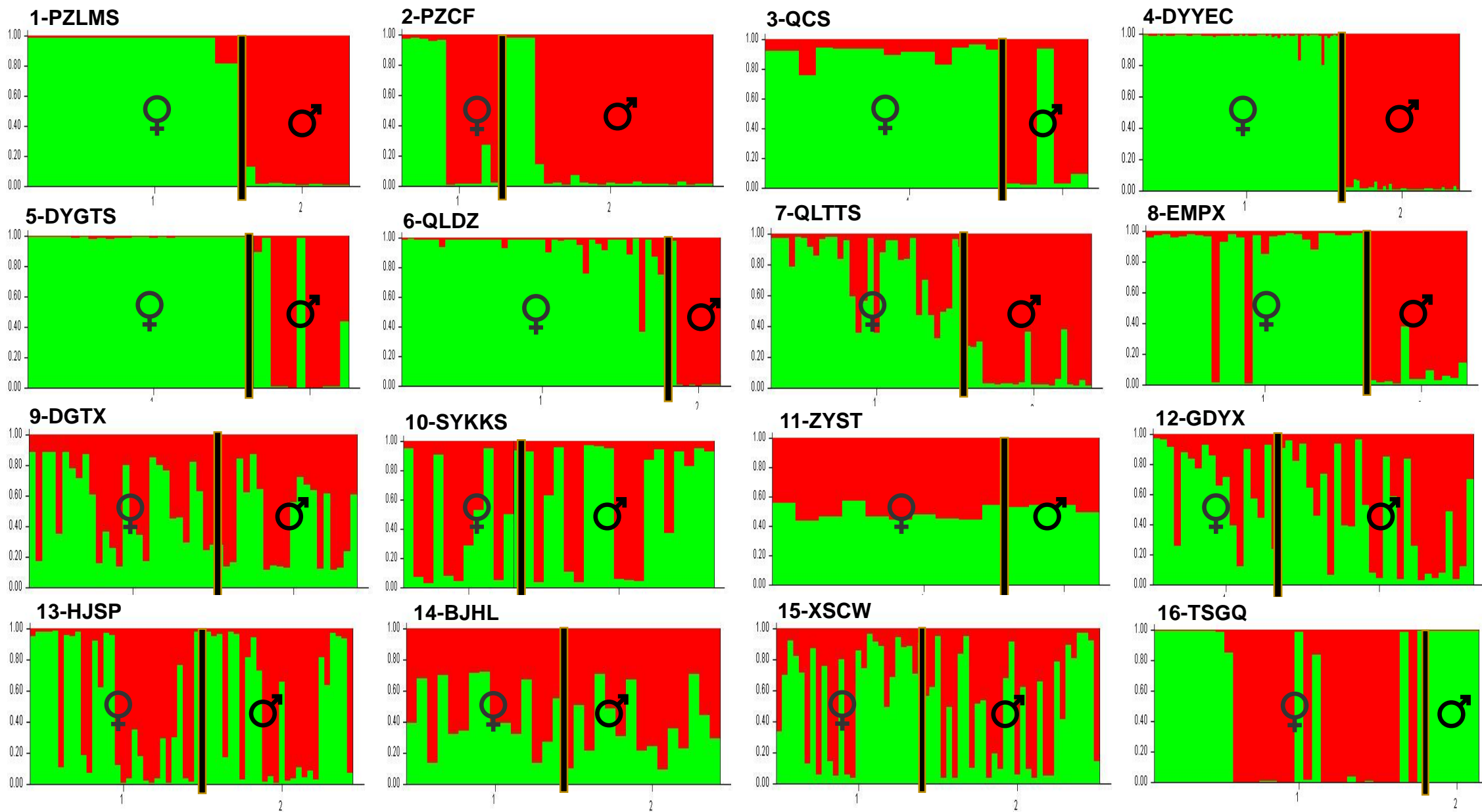

Supplement: FIGURE S2 — STRUCTURE analyses for two groups and per population. [file Image_2.PDF]

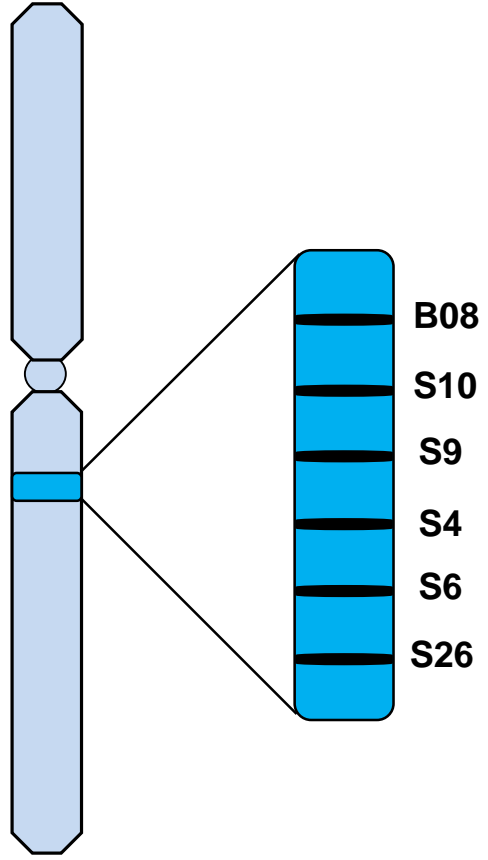

Supplement: FIGURE S3 — Relative position of sex-linked loci. [file Image_3.PDF]
