# Supplementary material for: Suppressed Recombination of Sex Chromosomes Is Not Caused by Chromosomal Reciprocal Translocation in Spiny Frog (Quasipaa boulengeri)
Source: Front Genet. 2018 Aug 27;9:288. doi: 10.3389/fgene.2018.00288 (PMC6119705; doi:10.3389/fgene.2018.00288)
Supplement: TABLE S2 — Primer details for microsatellite loci. [file Table_2.DOC]

**Table S2** Primer details for eleven microsatellite loci.

| **Locus** | **Primers (5’-3’; for/rev)** | **Repeat Motifs** | **Tm( °C)** | **Size (bp)** | **Location** |
| --- | --- | --- | --- | --- | --- |
| S4 | TGTAAAGAGTGCACAAACTGTTG GGATGGCTTTGTAAGTGATTGT | (AAT)_7_ | 62 | 92-134 | sex chromosome |
| S6 | TGAATTGTGATTGTAATGTCTTCC GGGAACTAGAGCATCTATGGTG | (TA)_4_(AAT)_6_ | 58 | 208-223 | sex chromosome |
| S9 | ACCAGACTTGCCTGAAGACTAC CGAGAAGCACTTGGAGAACTAT | (AAT)_11_(AAC)_5_ | 62 | 250-292 | sex chromosome |
| S10 | CCATTACCCTTCACTGTGAGAT AGGATATGCTGGGACTTTACAA | (AC)_8_ | 60 | 120-176 | sex chromosome |
| S26 | TAAGAGCTTCCTTACACCCA TAGTTGTAAAAGCAAACTGTCA | (AGAT)_14_ | 62 | 274-386 | sex chromosome |
| B08 | GCACGTACATTCATTCTGTG TACATACAAGTAGGGGGCTC | (AC)_9_ | 58 | 362-432 | sex chromosome |
| SSR02-D56 | CTTCAGTCCGGTTCTAGCTC | (GCT)5 | 64 | 243-255 | autosome |
|  | AGGACTTCACACACTGGATGC |  |  |  |  |
| SSR25-B12 | CTGCACCCTTACCACCACA | (AT)7 | 56 | 167-181 | autosome |
|  | GCCTCCATTTCAGTGTAGGT |  |  |  |  |
| QBb1 | CCTTACATTGGTTGTCCTCGTC | (TCTA)23 | 60 | 160-344 | autosome |
|  | AAGAGTGTCGCTGGGATCAGG |  |  |  |  |
| QBb5 | CTGCTTGCCTTTGTGTAAT | (TCTA)11 | 60 | 110-278 | autosome |
|  | ATCTCCAGGATAAAGTTGTG |  |  |  |  |
| QBb45 | GGGTTAGGTAAGGATAAGAGAT | (GATA)17 | 58 | 121-197 | autosome |
|  | ATCAGTAGGCAGCAGACATAC |  |  |  |  |
