# Supplementary material for: Suppressed Recombination of Sex Chromosomes Is Not Caused by Chromosomal Reciprocal Translocation in Spiny Frog (Quasipaa boulengeri)
Source: Front Genet. 2018 Aug 27;9:288. doi: 10.3389/fgene.2018.00288 (PMC6119705; doi:10.3389/fgene.2018.00288)
Supplement: TABLE S3 — Genetic diversity of sex-linked loci per population in western Sichuan Basin. HEY, expected heterozygosity for Y chromosome; HEX, expected heterozygosity for X chromosome; θY, genetic diversity index of Y chromosome; θX, genetic diversity index of X chromosome; E(θY), expected genetic diversity index of Y chromosome; E(θX), expected genetic diversity index of X chromosome. [file Table_3.DOC]

**Table S3** Genetic diversity of sex-linked loci per population in western Sichuan Basin.

| **Population** | **Locus** | **H_EY_** | **H_EX_** | **θ_Y_** | **θx** | **E(θ_Y_)** | **E(θ_X_)** |
| --- | --- | --- | --- | --- | --- | --- | --- |
| 1-PZLMS | S4 | 0.00 | 0.47 | 0.00 | 1.27 | 0.32 | 0.95 |
| 1-PZLMS | S6 | 0.00 | 0.47 | 0.00 | 1.30 | 0.33 | 0.98 |
| 1-PZLMS | S26 | 0.47 | 0.65 | 1.27 | 3.55 | 1.21 | 3.62 |
| 1-PZLMS | S9 | 0.00 | 0.47 | 0.00 | 1.27 | 0.32 | 0.95 |
| 1-PZLMS | S10 | 0.00 | 0.47 | 0.00 | 1.27 | 0.32 | 0.95 |
| 1-PZLMS | B08 | 0.00 | 0.63 | 0.00 | 3.08 | 0.77 | 2.31 |
| 2-PZCF | S4 | 0.00 | 0.31 | 0.00 | 0.56 | 0.14 | 0.42 |
| 2-PZCF | S6 | 0.00 | 0.31 | 0.00 | 0.56 | 0.14 | 0.42 |
| 2-PZCF | S26 | 0.56 | 0.80 | 2.07 | 11.95 | 3.51 | 10.52 |
| 2-PZCF | S9 | 0.00 | 0.31 | 0.00 | 0.56 | 0.14 | 0.42 |
| 2-PZCF | S10 | 0.00 | 0.31 | 0.00 | 0.56 | 0.14 | 0.42 |
| 2-PZCF | B08 | 0.00 | 0.57 | 0.00 | 2.20 | 0.55 | 1.65 |
| 3-QCS | S4 | 0.00 | 0.50 | 0.00 | 1.49 | 0.37 | 1.11 |
| 3-QCS | S6 | 0.00 | 0.46 | 0.00 | 1.19 | 0.30 | 0.90 |
| 3-QCS | S26 | 0.44 | 0.60 | 1.12 | 2.58 | 0.92 | 2.77 |
| 3-QCS | S9 | 0.00 | 0.71 | 0.00 | 5.64 | 1.41 | 4.23 |
| 3-QCS | S10 | 0.00 | 0.50 | 0.00 | 1.49 | 0.37 | 1.11 |
| 3-QCS | B08 | 0.00 | 0.53 | 0.00 | 1.74 | 0.44 | 1.31 |
| 4-YEC | S4 | 0.00 | 0.36 | 0.00 | 0.73 | 0.18 | 0.55 |
| 4-YEC | S6 | 0.00 | 0.36 | 0.00 | 0.72 | 0.18 | 0.54 |
| 4-YEC | S9 | 0.00 | 0.68 | 0.00 | 4.46 | 1.11 | 3.34 |
| 4-YEC | S10 | 0.00 | 0.37 | 0.00 | 0.74 | 0.19 | 0.56 |
| 4-YEC | B08 | 0.00 | 0.37 | 0.00 | 0.76 | 0.19 | 0.57 |
| 5-GTS | S4 | 0.00 | 0.50 | 0.00 | 1.47 | 0.37 | 1.10 |
| 5-GTS | S6 | 0.00 | 0.37 | 0.00 | 0.75 | 0.19 | 0.56 |
| 5-GTS | S26 | 0.22 | 0.64 | 0.32 | 3.35 | 0.92 | 2.75 |
| 5-GTS | S9 | 0.00 | 0.50 | 0.00 | 1.46 | 0.37 | 1.10 |
| 5-GTS | S10 | 0.00 | 0.50 | 0.00 | 1.47 | 0.37 | 1.10 |
| 5-GTS | B08 | 0.00 | 0.50 | 0.00 | 1.47 | 0.37 | 1.10 |
| 6-QLDZ | S4 | 0.00 | 0.48 | 0.00 | 1.31 | 0.33 | 0.99 |
| 6-QLDZ | S9 | 0.00 | 0.55 | 0.00 | 2.00 | 0.50 | 1.50 |
| 6-QLDZ | S10 | 0.00 | 0.66 | 0.00 | 3.91 | 0.98 | 2.94 |
| 6-QLDZ | B08 | 0.18 | 0.46 | 0.24 | 1.23 | 0.37 | 1.10 |
| 7-QLTTS | S4 | 0.00 | 0.68 | 0.00 | 4.44 | 1.11 | 3.33 |
| 7-QLTTS | S9 | 0.00 | 0.69 | 0.00 | 4.77 | 1.19 | 3.58 |
| 7-QLTTS | S10 | 0.00 | 0.40 | 0.00 | 0.90 | 0.22 | 0.67 |
| 7-QLTTS | B08 | 0.30 | 0.43 | 0.51 | 1.05 | 0.39 | 1.17 |
| 8-EMPX | S4 | 0.00 | 0.52 | 0.00 | 1.64 | 0.41 | 1.23 |
| 8-EMPX | S9 | 0.00 | 0.67 | 0.00 | 4.00 | 1.00 | 3.00 |
| 8-EMPX | S10 | 0.00 | 0.43 | 0.00 | 1.04 | 0.26 | 0.78 |
| 8-EMPX | B08 | 0.00 | 0.44 | 0.00 | 1.10 | 0.27 | 0.82 |
