# Supplementary material for: Suppressed Recombination of Sex Chromosomes Is Not Caused by Chromosomal Reciprocal Translocation in Spiny Frog (Quasipaa boulengeri)
Source: Front Genet. 2018 Aug 27;9:288. doi: 10.3389/fgene.2018.00288 (PMC6119705; doi:10.3389/fgene.2018.00288)
Supplement: TABLE S4 — P-values for pairwise linkage disequilibrium analyses among sex-linked loci. [file Table_4.DOC]

**Table S4** P-values for pairwise linkage disequilibrium analyses among sex-linked loci.

| **Loci** | **P values** |
| --- | --- |
| S4&S6 | 0.03 |
| S4&S26 | 0.24 |
| S6&S26 | 0.00 |
| S4&S9 | 0.00 |
| S6&S9 | 0.00 |
| S26&S9 | 0.13 |
| S4&S10 | 0.21 |
| S6&S10 | 0.82 |
| S26&S10 | 0.16 |
| S9&S10 | 0.00 |
| S4&B08 | 0.35 |
| S6&B08 | 0.84 |
| S26&B08 | 0.16 |
| S9&B08 | 0.10 |
| S10&B08 | 0.12 |
